# Uncertainty-aware and interpretable evaluation of Cas9–gRNA and Cas12a–gRNA specificity for fully matched and partially mismatched targets with Deep Kernel Learning

**DOI:** 10.1093/nar/gkab1065

**Published:** 2021-11-17

**Authors:** Bogdan Kirillov, Ekaterina Savitskaya, Maxim Panov, Aleksey Y Ogurtsov, Svetlana A Shabalina, Eugene V Koonin, Konstantin V Severinov

**Affiliations:** Center for Life Sciences, Skolkovo Institute of Science and Technology, Moscow 143026, Russia; Center for Precision Genome Editing and Genetic Technologies for Biomedicine, Institute of Gene Biology, Russian Academy of Sciences, Moscow 119334, Russia; Center for Life Sciences, Skolkovo Institute of Science and Technology, Moscow 143026, Russia; Center for Computational and Data-Intensive Science and Engineering, Skolkovo Institute of Science and Technology, Moscow 143026, Russia; National Center for Biotechnology Information, National Library of Medicine, National Institutes of Health, Bethesda, MD 20894, USA; National Center for Biotechnology Information, National Library of Medicine, National Institutes of Health, Bethesda, MD 20894, USA; National Center for Biotechnology Information, National Library of Medicine, National Institutes of Health, Bethesda, MD 20894, USA; Center for Life Sciences, Skolkovo Institute of Science and Technology, Moscow 143026, Russia; Center for Precision Genome Editing and Genetic Technologies for Biomedicine, Institute of Gene Biology, Russian Academy of Sciences, Moscow 119334, Russia; Institute of Molecular Genetics, Russian Academy of Sciences, Moscow 123182, Russia; Waksman Institute for Microbiology, Rutgers, The State University of New Jersey, Piscataway, NJ 08854, USA

## Abstract

The choice of guide RNA (gRNA) for CRISPR-based gene targeting is an essential step in gene editing applications, but the prediction of gRNA specificity remains challenging. Lack of transparency and focus on point estimates of efficiency disregarding the information on possible error sources in the model limit the power of existing Deep Learning-based methods. To overcome these problems, we present a new approach, a hybrid of Capsule Networks and Gaussian Processes. Our method predicts the cleavage efficiency of a gRNA with a corresponding confidence interval, which allows the user to incorporate information regarding possible model errors into the experimental design. We provide the first utilization of uncertainty estimation in computational gRNA design, which is a critical step toward accurate decision-making for future CRISPR applications. The proposed solution demonstrates acceptable confidence intervals for most test sets and shows regression quality similar to existing models. We introduce a set of criteria for gRNA selection based on off-target cleavage efficiency and its variance and present a collection of pre-computed gRNAs for human chromosome 22. Using Neural Network Interpretation methods, we show that our model rediscovers an established biological factor underlying cleavage efficiency, the importance of the seed region in gRNA.

## INTRODUCTION

Prokaryotic Class 2 CRISPR (Clustered Regularly Interspaced Short Palindromic Repeats)–Cas (CRISPR-associated) systems possess effector modules that consist of a single multi-domain RNA-Guided Nuclease (RGN) (1–3), which makes them an invaluable resource for developing tools for genome editing and other applications, such as highly sensitive nucleic acid detection (4). In these applications, an RGN functions by binding, in many cases, with subsequent cleavage, the target DNA or RNA at programmed sites through sequence-specific base-pairing between the unique portion of the guide RNAs (gRNAs), known as a spacer, and the complementary sequence in DNA or RNA targets (1,5,6). From the experimental standpoint, it is hard to distinguish between binding and cleavage efficiency for the experimental data do not offer the distinction between the case where the binding happened, but cleavage has not, and the case where binding did not occur. Therefore, in this study we use the term ‘cleavage efficiency’ even in the cases where there is no actual cleavage, such as dCas9 gene expression modulation experiments. The strength of base-pairing interactions can modulate the RGN binding affinity and reduce off-target effects (7,8). Improvement in specificity, on-target cleavage efficiency, and reduction of off-target cleavage can be achieved through directed engineering of the RGN or sgRNA, as well as modification of Cas-sgRNA delivery methods. Improvements in each of these directions are crucial for the full realization of the enormous potential of the RGN-based technologies (7–9).

A major challenge of CRISPR-based genome engineering techniques is accurate prediction or estimation/evaluation of the gRNA specificity for the target. It has been shown that gRNAs widely differ in the efficiency of their interaction with fully matching targets. In some cases, interactions between the gRNA and the target tolerate up to several mismatches, resulting in off-target effects that are difficult to predict (10,11). Both the on-target cleavage efficiency and off-target effects have to be taken into consideration when developing gRNAs for biological and especially for health-related applications. Several large-scale studies have been conducted aiming at the determination of the molecular features that determine gRNA specificity *in vivo* (12–16). Notwithstanding differences in details, all these studies postulated that for popular Cas9 RGN intermediate GC content, a G in the proximal position of the PAM (Protospacer Adjacent Motif) and a C in the first PAM position were the main prerequisites of efficient targeting. Despite some inconsistencies between the results of these analyses, they were implemented in predictive models for binary classification or/and regression analysis of on-target vs off-target specificity (Guide Picker (17), Azimuth (https://github.com/MicrosoftResearch/Azimuth), WU-CRISPR (18), CRISPRscan (15)). Comparison of the performance of these tools (19,20) has shown that sequence-based off-target predictions were highly reliable, identifying most off-targets with cleavage frequency >0.1%, whereas the false positive rate can be substantially reduced using a cutoff on the output of off-target model (21). Additionally, it has been shown that the optimal on-target efficiency prediction model strongly depends on whether the gRNA is expressed from the U6 RNA polymerase III promoter that is routinely used for small RNA production or transcribed in vitro followed by the introduction of pre-programmed RGN inside cells (19). Nevertheless, the best prediction approaches can substantially reduce the time spent on gRNA screening.

Deep learning allows design of optimal gRNAs without any prior assumptions on the structural features of the target region. Several tools exploiting deep learning have been recently developed for predicting the on-target cleavage efficiency of Cas9 and Cas12a nucleases (DeepCRISPR (22), DeepCpf1 (23)) and the off-target effects of Cas9 (24). These tools exhibited high performance in on-target and off-target predictions of gRNAs, allowing one to design optimal gRNA for custom applications. However, the tools provide only part of the relevant information for experimental design, namely, a point estimate of the on-target and off-target cleavage efficiencies—a single digit that estimates the cleavage efficiency of gRNA. Because cleavage efficiency varies between replications of the experiment, estimating the parameters (mean and variance) of cleavage efficiency distribution is preferable to predicting a single value, as it will allow one to incorporate information on prediction error in the decision-making process.

Here, we describe a new approach for prediction of gRNA on-target cleavage efficiency and off-target probability for various Cas9 proteins and AsCas12a, a Cas12a RGN from *Acida minococcus* sp.). Our method is based on a combination of a Hit-Or-Miss capsule network (introduced in arXiv preprint arXiv:1806.06519, 2018), which acts as a feature extractor, and a Gaussian Process to model the distribution from which the real-valued on-target and off-target activities are sampled. This approach allows estimation of the cleavage efficiency of gRNAs containing mismatches to their target sites using large-scale measurements across multiple cell models from recent studies (25) as training sets. Although our setup is quite different from the common applications of regression, we show that our tool performs as well or better compared with previous Deep Learning attempts in regression tasks. Moreover, for the first time, we introduce uncertainty quantification in the CRISPR cleavage efficiency estimation pipeline. Our tool provides the end user with the ability to carefully assess the deviation in cleavage efficiency estimates in order to make optimal decisions during CRISPR experiment design. For example, the tool helps select the guides that not only have the highest predicted on-target cleavage efficiency, but also those with the most confident predictions. This feature of the method can improve the quality of experimental design by providing information on possible model error.

## MATERIALS AND METHODS

### Problem setup

We use the models to predict cleavage efficiency from sequences. Cleavage efficiency is a real number usually ranging from 0 to 1. It can be slightly greater than 1 or smaller than 0 (depends on the dataset), but for the purpose of prediction, we scale and shift the labels so the maximum is 1 and minimum is 0. The sequences are strings over an alphabet of four symbols (A, T, G and C), 20–23 symbols long. The problem is the task of regression which we solve using a neural network-enhanced Gaussian process. We provide the solution that, unlike others, not only gives a point estimate of the cleavage efficiency, but, for the first time, also gives the confidence interval for it.

### Data collection and preprocessing

For on-target Cas9 cleavage efficiency prediction, we used three non-overlapping datasets, geCRISPR, DeepCRISPR and DeepHF. The geCRISPR dataset (26) consists of 4569 experimentally verified gRNAs for Cas9 derived from *Homo sapiens*, *Danio rerio*, *Mus musculus* and *Xenopus tropicalis*. DeepCRISPR dataset (22) consists of 16 492 experimentally verified gRNAs for Cas9 derived from four human cell lines (hela, hl60, hct116, hek239t). DeepHF (27) provides the data for SpCas9 (55604 sequences) and two high fidelity orthologs: SpCas9HF1 (56888 sequences) and eSpCas9 (58617 sequences). For prediction of Cas9 efficiency with mismatched gRNA and target sequences, we used the dataset of Jost et al (25) with 26 248 gRNA–target pairs. For on-target Cas12a cleavage efficiency prediction, we used the DeepCpf1 (23) dataset that consist of 20 506 experimentally verified gRNAs for Cas12a. For Cas12a off-target prediction, we used the dataset of (28) with 1565 gRNA–target pairs. Before training the neural networks, we used one-hot encoding on every sequence in the on-target datasets, as shown on Figure 2A. For the off-target datasets, the encoding algorithm was used twice, that is, once on the guide, and the second time, on the target, so that the output is a two-channel image (2, 4, *N*) where *N* is the length of the input sequences. For the on-target datasets, the algorithm was applied only once, so that the output is a (4, *N*) image.

### Overview of the GuideHOM architecture

We developed a deep kernel learning model, named GuideHOM (Guide Hit-Or-Miss). GuideHOM employs a Hit-Or-Miss capsule network as an intermediate feature extractor built upon a preprocessing module and a Gaussian Process to estimate the distribution of the cleavage efficiency of RGN programmed with a given gRNA. The estimation is based on the representation produced by the Hit-Or-Miss capsules to predict on-target and off-target cleavage efficiency using gRNA spacer sequence features (see Figure 2A for example of a single input encoding). In GuideHOM, we implemented two preprocessing approaches that differed in terms of the prediction strategy and the analyzed datasets. The first approach predicts the on-target cleavage efficiency based on the gRNA spacer sequence only, under the assumption that the target sequence is its exact complement. The second approach aims to estimate the cleavage efficiency based on the mismatching gRNA spacer-target pairs. We assume that all sequences in the genome that match a particular gRNA are cleaved with the same efficiency although this is unlikely to hold precisely, for example, due to differences in chromatin accessibility. However, in this work, we do not use chromatin accessibility or data on other potential contributing factors, relying solely on the gRNA and target sequences.

**Figure 1.**
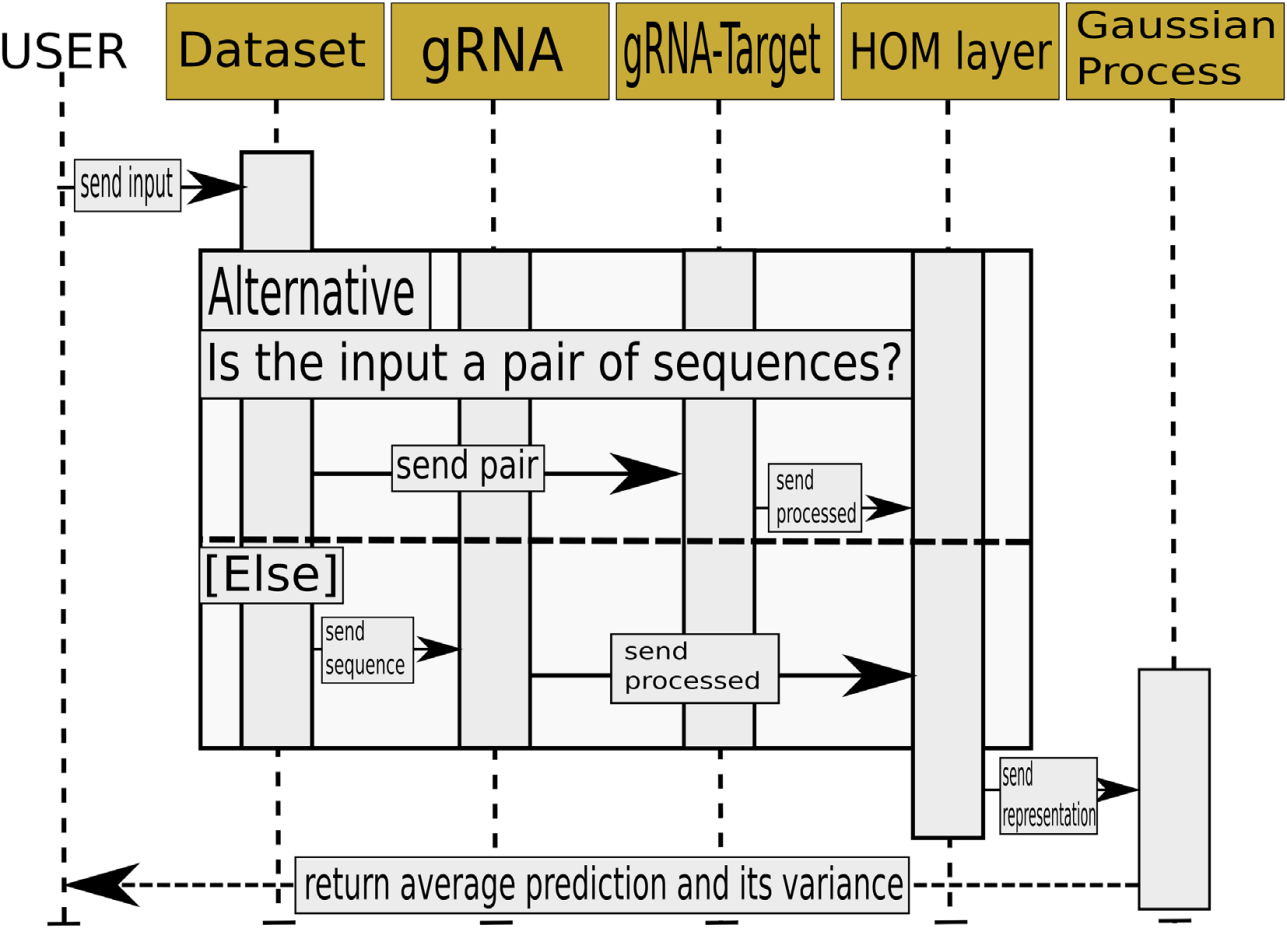
The UML (Unified Modelling Language) sequence diagram of a single input use case for the GuideHOM architecture. First, the user supplies the model with an input through the Dataset object. The Dataset object supplies the preselected preprocessing module with one-hot encoded sequence or pair. Either of the preprocessing modules supplies the HOM capsule layer with the preprocessing output. The HOM capsule layer computes coordinates of gRNA/gRNA pairs in the guide space, then, sends the coordinates to the Gaussian Process. The Gaussian Process samples activities from the approximate distribution it has learnt, computes the mean and variance, then, sends the outputs back to the user.

**Figure 2.**
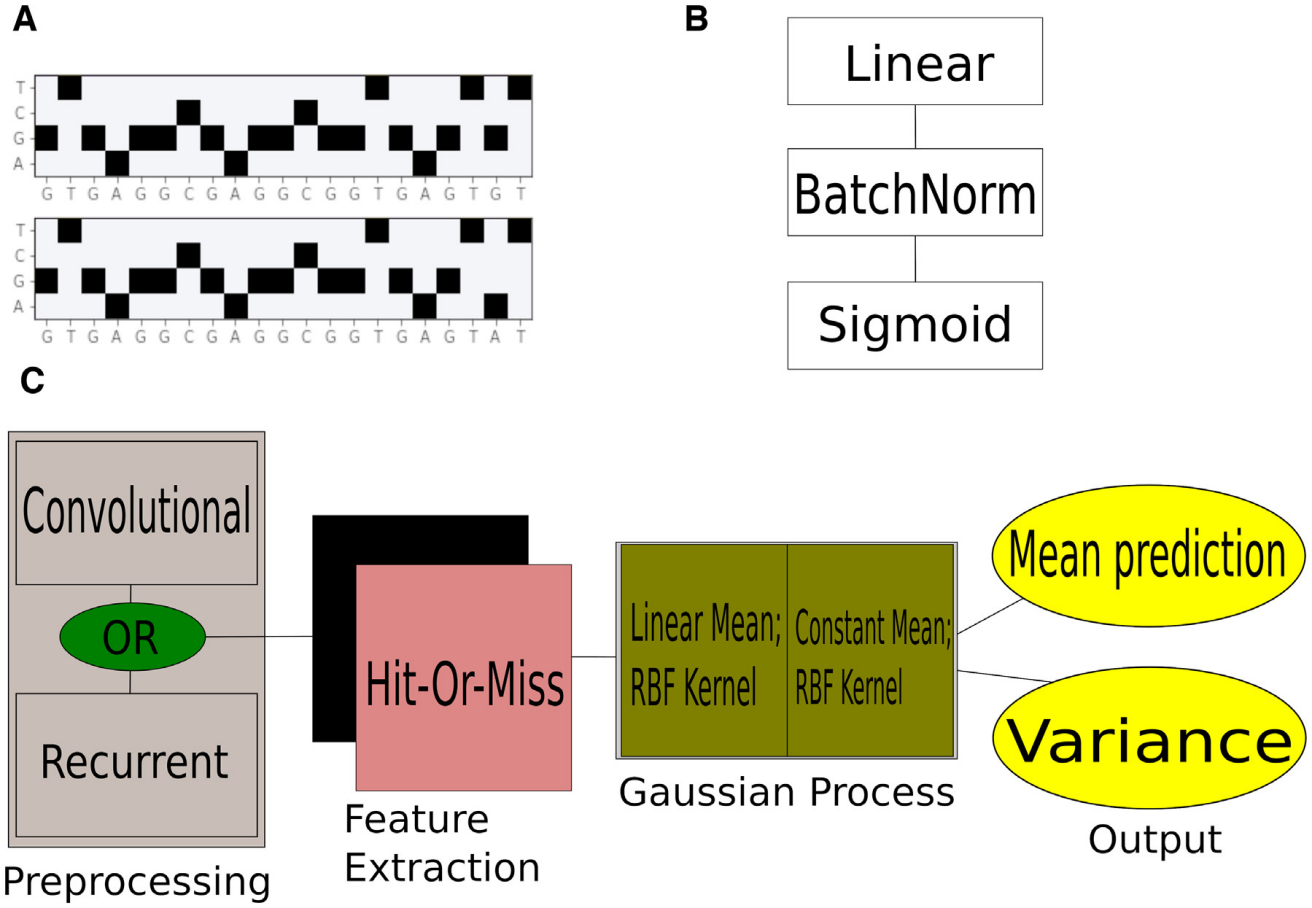
The GuideHOM architecture. (**A**) The input: one or two one-hot encoded sequences. To predict on-target cleavage efficiency based only on gRNA, we use the 1D convolutional or recurrent preprocessing modules, and to predict cleavage efficiency based on mismatched gRNA and target sequences, we use 2D convolutional preprocessing. (**B**) The structure of Hit-or-Miss capsule layer; 5 capsule layers are used in the Hit-or-Miss network. (**C**) Schematic illustration of the GuideHOM architecture. See ‘Materials and Methods’ for more details.

The model is a deep neural network that consists of three modules: (i) preprocessing subnetwork that extracts low-level sequence features using either a 1D or 2D convolutional layer followed by Leaky ReLU (the Rectified Linear Unit, a commonly used activation function) or an LSTM layer (Long Short Term Memory networks); (ii) encoder subnetwork that extracts high-level sequence features from preprocessed inputs using a set of Hit-or-Miss capsules (29), and (iii) a set of Gaussian Processes (30) that estimate cleavage efficiency based on the extracted features computed by the encoder subnetwork (Figure 2C). The sequences are encoded in an one-hot fashion as shown on Figure 2A: each nucleotide *i* is replaced by a vector of 4 components with the i-th component being equal to 1 and every other component set to zero. For gRNA preprocessing, the final, machine-readable input is an array of size (4, *N*), where N is the length of gRNA spacer; for gRNA-target preprocessing, the final input is an array of size (2, 4, *N*). To predict on-target cleavage efficiency based only on gRNA, we use the 1D convolutional or recurrent preprocessing modules. To predict cleavage on- and off-target efficiencies based on gRNA and target sequences we use 2D convolutional preprocessing. For a gRNA–target pair, our model estimates the lower and upper bounds of the cleavage efficiency. The recurrent layer is not used for the case with two sequence inputs, in order to minimize unnecessary complexity in the setup. Pytorch (31) is used as the deep learning framework and GPytorch (32) is used as the Gaussian Process library. All experiments were carried out with a single Nvidia GeForce 1070 GPU. The same architecture and training routine were used for all datasets with minor modifications to accommodate different lengths of the input data (for example, the geCRISPR dataset provides gRNAs with 20 nucleotide spacer lengths only, compared to DeepCRISPR, DeepHF and DeepCpf1 that provide 23nt). The simplest use case of the GuideHOM model is shown in Figure 1.

As can be seen from Figure 1, the choice between gRNA and gRNA–target preprocessing module depends only on the input data in the dataset, whereas the rest of the workflow remains the same: preprocessing, computation of HOM representations, sampling the cleavage efficiency distribution, computation of mean cleavage efficiency and its variance. The general architecture is shown in Figure 2B and C, and the dataset-specific changes are described in Supplementary Table S1.

### Preprocessing subnetwork

The preprocessing subnetwork provides the extraction of low-level sequence features on the level of k-mers. For the experiments in this work, we used three types of preprocessing subnetworks: (i) 1D convolutional, (ii) 2D convolutional and (iii) LSTM-based. An 1D convolutional preprocessing subnetwork consists of a 1d CoordConv layer (33) followed by Leaky ReLU activation. The 2D convolutional layer is the same but with 2D convolutions. We use CoordConv instead of convolutions because it is shown to work better on one-hot encoded data (33). The LSTM preprocessing consists of four consecutive LSTM layers.

### Encoder subnetwork for feature extraction

The encoder is used to learn patterns based on the sets of k-mers and to present the learned patterns in a concise manner as a matrix of real numbers. The encoder consists of a number of Hit-Or-Miss capsules applied to the preprocessed input in parallel. Each Hit-Or-Miss capsule is a linear layer followed by batch normalization to accelerate the learning process, and sigmoid activation to constrain the output between 0 and 1 (Figure 2B). Hit-Or-Miss capsule *i* encodes the difference between an input example and a center of the space of possible outputs:

$$\begin{equation*} O_i(X) = C - Sigmoid(BatchNorm_i(Linear_i(X))), \end{equation*}$$

where *C* = [0.5, 0.5, ...0.5]. In case of classification, when Hit-or-Miss capsules are optimized with their special Centripetal loss, the perfect ‘hit’ is the vector filled with zeros, and the wrong answers accumulate large ‘misses’. In our case, we did not use the classification (centripetal) loss and classification setup, so the ‘misses’ simply encode the position of the input in the space of all possible inputs, not necessarily the class vectors. Consult the Supplementary Table S1 for details for the structure and parameters used in the encoder subnetwork.

### Gaussian process for cleavage efficiency prediction

For prediction of the cleavage efficiency, we use a model called Gaussian Process (GP). A Gaussian process is a probability distribution over possible functions that fit a set of points:

$$\begin{equation*} f(x) = GP(m(x), k(x, x^{\prime })), \end{equation*}$$

where *x* and *x*′ are the pair of inputs, *f*(*x*) is the function we would like to fit, *m*(*x*) - mean function and *k*(*x*, *x*′) - covariance function, such as:

$$\begin{equation*} m(x) = {\bf E}[f (x)], \end{equation*}$$

$$\begin{equation*} k(x, x^{\prime }) = {\bf E}[(f (x) - m(x))(f (x^{\prime }) - m(x^{\prime }))]. \end{equation*}$$

Mean and covariance functions denote the priors of the distribution over functions. By setting mean and covariance, we choose a set of functions that are used for inference. We fit the Gaussian Process by selecting from a prior distribution only those functions that agree with the observations. This is achieved via optimizing the parameters of the covariance function, the mean function and the encoder neural network using gradient descent. We obtain actual predictions of a value by sampling the Gaussian Process. Covariance function specifies the covariance between pairs of random variables and mean function specifies the base level of the predicted value. As our loss function we use Evidence Lower Bound (34):

$$\begin{equation*} ELBO(q(x)) = {\bf E}[\log p(x|z)] - {\bf KL}(q(z)||p(z)), \end{equation*}$$

where *x* is the observed cleavage efficiency, $z$ is the latent variable (representation computed by the neural network), *p*(*x*|$z$) is the conditional distribution of cleavage efficiency given the latent variable, *q*($z$) is the variational distribution of the latent variable, *p*($z$) is the prior distribution of the latent variable, KL is their Kullback–Leibler divergence. It is a ‘natural’ loss function for deep Gaussian processes that, in our case, can be efficiently computed and optimized via gradient descent using GPytorch primitives. For the gradient descent, we use the Adam algorithm introduced in arXiv preprint arXiv:1412.6980, 2014 with starting learning rate λ_0_ = 0.01. We schedule our learning rate to decrease by multiplying it by 0.9 every tenth epoch. The model is trained for 60 epochs. Such step wise reduction helps in convergence towards good local minima of the loss function surface. We also experiment with additional, more traditional loss, Mean Squared Error, so for a number of experiments (in the Supplementary Table S3 and everywhere else referred as ‘E+M’), the loss is as follows:

$$\begin{equation*} Loss_{E+M}(q(x), y, \hat{y}) = ELBO(q(x)) + \alpha MSE(y, \hat{y}), \end{equation*}$$

where *y* is the optimization target, $\hat{y}$ is the prediction, that is, the mean of a sample from GP and α = 0.5. Gaussian Processes can be nested just as linear layers in neural networks. Our network consists of two layers with a linear and constant mean. The output shape of the first layer is 2, and the second process used the previous output as input and outputs the 1D distribution. The scheme is similar to a fully connected neural network, but outputs not a single number but a distribution that can be sampled. After the sampling, we compute the mean prediction and the confidence interval, which is the uncertainty-aware prediction of cleavage efficiency we aim at obtaining. Agglomerative clustering of the guide space for motif analysis was performed using SciPy library (35), with default settings, max_d of 2 and criterion ‘distance’. Weblogo (36) library was used to visualize motifs.

### Performance metrics for regression, quality of confidence intervals and cross validation

To analyze the performance of the regression models, we use three classical metrics - determination coefficient (*r*^2^), Pearson and Spearman correlation coefficients (PCC and SCC respectively), computed using scipy library. To understand whether the confidence intervals the model gives is acceptable, we compute how many real labels for examples from the test set lie between the predicted label and predicted standard deviation:

$$\begin{equation*} \rho (Y, \hat{Y}, \sigma , M) = \frac{\sum _{i=0}^N I(Y_i \in [\hat{Y_i}-M\sigma _i, \hat{Y_i}+M\sigma _i)}{N}, \end{equation*}$$

where *N* is the test set size, *I* is the indicator function which is 1 if the argument is true and 0 othervise, *M* ∈ [1, 2, 3]. We compute ρ for one, two and three standard deviations and check whether the values are close to 68%, 95% and 99.7%, respectively. We denote those values as ρ_68_, ρ_95_ and ρ_99.7_.

To robustly assess the performance of the models, in addition to hold-out set testing used in most previous studies (23,25,27), 10-fold cross validation was performed: the data was randomly split into 10-fold, the model was trained on 9-fold, the performance was tested on the 10th and the results were saved, The results of 10 performance tests were next used to compute the mean performance and standard deviation. These results are reported separately from the results obtained with the hold-out set.

### Neural network interpretation

To interpret the predictions of the model, the Accumulated Local Effects (ALE, (37)) are computed using Python library alibi (38). ALEs are computed over *M* randomly generated synthetic gRNAs (or gRNA-target pairs, depends on the model) that are flattened into (*M*, 4*N*) or (*M*, 4 × 2*N*) matrix. The model requires (*M*, 2, 4, *N*) or (*M*, 4, *N*), where *N* is the length of the input, so an intermediate class to reshape the inputs is used. *M* in our work is set to 10 000. The resulting ALE values have the shape of (4*N*, *A*, *B*), where *A* is the number of feature intervals, in our case, 2 for 0 and 1 of one-hot encoded nucleotides, and *B* is the number of prediction targets, again, 2 in our case, corresponding to mean efficiency and variance. We are interested only in the component of features that corresponds to the values of 1, because we would like to see the importance of a presence of a certain nucleotide on a certain position.

The heatmaps from Figure 7, Supplementary Figures S1 and S2, are constructed from the ALE explainer class, first and second components of the vector that corresponds to the value 1 of features for mean efficiency and variance respectively. See the Figure 7.ipynb, Supplementary Figure1.ipynb, Supplementary Figure 2.ipynb and reproduce_explanations.py notebooks and scripts.

For logo sequences, constructed out of ALE values, the softmax with additional temperature parameter is calculated:

$$\begin{equation*} y_i(X, t) = \frac{\exp (\frac{X_i}{t})}{\sum _{j=1}^N \exp (\frac{X_j}{t})}, \end{equation*}$$

where *t* is temperature parameter, the less temperature is, the more distinct the output probabilities are, *X*_*i*_ are the features that correspond to *i*th nucleotide out of four, *i* and *j* are the counters. Softmax is applied along the nucleotide axis, the resulting matrix is of (4, *N*) size, and if we sum the vector along the first axis, we get the vector of *N* ones. We use weblogo to draw the resulting sequences.

### The genome analysis pipeline

Input: the raw sequence data, downloaded from the UCSC genome browser and respective annotation. Output: the list of possible gRNAs with computed average cleavage efficiency, standard deviation. The input is downloaded with the following options: Chromosome - Download FASTA, Visible range (while whole chromosome is opened in the browser); Annotation – Download csv. The pipeline proceeds as follows:

- the genes are extracted from the chromosome sequence according to the annotation using a BioPython-based script;
- Cas-OFFinder (39) is used for each separate gene.fasta to produce a list of potential targets. The mask is NNNNNNNNNNNNNNNNNNNNNRG for Cas9, TTTNNNNNNNNNNNNNNNNNNNNN for Cas12a. The result is saved in .tsv files, one for each gene;
- the model is used for each Cas-OFFinder output. For each target sequence, the model computes the average cleavage efficiency and standard deviation (or just the cleavage efficiency in case of (25)). If the model requires two sequences, the input target is duplicated to form (4, *N*, 2) vector, so the input sequence acts as both gRNA and target;
- the guides are sorted by cleavage efficiency in descending order. Each result is saved in a table that combines Cas-OFFinder output and model output.

For this analysis we use the best performing Cas9 and Cas12a on-target models. The results are available at a dedicated Zenodo repository, consult the ‘Code and data availability’ section for details.

## RESULTS

### GuideHOM provides acceptable confidence intervals and accurate and reliable predictions of on-target cleavage efficiency

In our study, combination of Hit-Or-Miss networks (arXiv preprint arXiv:1806.06519, 2018) and Deep Gaussian Processes (30) helps to augment the point estimates of cleavage efficiency with the information on prediction uncertainty. To our knowledge, this is the first application of uncertainty-aware machine learning for CRISPR cleavage prediction efficiency. Using the confidence intervals derived from the model helps to overcome the problem of noisy and biased training data. We attempt to replicate the results of each original study from which we extracted datasets used to train the GuideHOM model. It allows us to systematically compare its performance with the original models.

**Figure 3.**
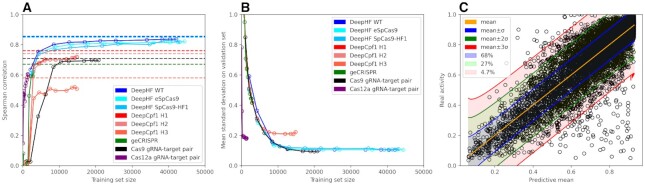
Models learning curves on different datasets. (**A**) Learning curves for indicated models/datasets are shown. Dashed lines indicate the performance of previous tools (DeepHF, DeepCpf1 and so on). The line for Cas12a off-target dataset is not shown since only classification models for this dataset are published. (**B**) Reduction of uncertainty dependent on the training set size. The models for DeepHF and DeepCpf1 shown here are CNN-based and were trained to minimize ELBO+MSE. The Cas9 gRNA-target pair model was also trained to minimize ELBO+MSE. (**C**) The output of trained model. The dots denote examples from the validation set. For each input example, the model outputs a sample of predictions, the mean of which is shown as the orange line, and the standard deviation gives the possible range of errors. 68% of all real activities lie in the blue area between the orange and blue lines—for 68% examples, the real cleavage efficiency is no more than one predicted standard deviation larger or smaller than the predicted mean cleavage efficiency. 27% of real activities lie in the green area—only 27% of examples have the real cleavage efficiency larger or smaller than the predicted mean cleavage efficiency for more than one predicted standard deviation. 4.7% of real activities lie in the red area—for 4.7% examples, the real cleavage efficiency is larger or smaller than the predicted mean cleavage efficiency for more than two predicted standard deviations. The rest 0.3% are the ones that have the real cleavage efficiency larger or smaller than the predicted mean cleavage efficiency for more than three predicted standard deviations.

We use the same data for training and testing in cases where the actual training and testing sets are available and the same train-test split ratio in other cases. Supplementary Table S2 shows the train-test splits corresponding to each analyzed dataset. The confidence intervals GuideHOM provides tend to be acceptable (see Figure 3C for visual explanation): the frequency of the real value *Y* present within the confidence intervals (with confidence level α) computed on the corresponding test sets was, for all cases, close to the preset confidence level (α = σ, 2σ, 3σ). As the loss function, we use ELBO (Evidence Lower Bound (34)) or a sum of ELBO and a half of MSE (Mean Squared Error). For example, the frequencies of the real values in *Y*_*mean*_ ± σ, *Y*_*mean*_ ± 2σ, *Y*_*mean*_ ± 3σ for a model trained with minimizing ELBO as an optimization objective are 0.7535, 0.9422 and 0.9835, respectively, as estimated on the respective validation set. The full table of confidence intervals for all trained models is given in Supplementary Table S3. According to the learning curves (Figure 3A), only about 30% of the DeepHF (27) and Cas9 gRNA-target pair (25) datasets provide sufficient data for the model to reach results comparable to the original models. The Cas9 gRNA-target pair model reaches lower levels of uncertainty while having less data to train on, which implies a lower noise level in Jost *et al.* (25) dataset (Figure 3B) because most of the uncertainty in the output comes from label noise in the dataset (arXiv preprint arXiv:1705.10694, 2017, 40).

The same performance dynamics is observed for all analyzed datasets: after the training sample size grows to about 10 000 gRNAs, the performance converges at a plateau so that the additional improvement is negligible. Uncertainty reduction follows a similar course: the smallest mean standard deviation is achieved near the 10,000 gRNAs mark and remains approximately constant with larger training sets. These observations suggest that for this version of the GuideHOM architecture, collecting datasets of more than 10 000 gRNAs yields diminishing returns in terms of the model accuracy, so a better strategy is to focus on the reduction of the label noise. However, the cut-off may have to be re-accessed depending on the type of gRNA library used, i.e. those targeting intronic, exonic regions, high/low expressed genes etc. The performance of the method for on-target cleavage efficiency prediction on benchmark datasets tends to be close to that of the best original point estimate models (Table 1; GuideHOM models are denoted as follows: preprocessing module (C for CNN or R for RNN), loss function (E for ELBO or E+M for ELBO+MSE); see the ‘Overview of GuideHOM architecture’, ‘Preprocessing subnetwork’ and ‘Gaussian process for cleavage efficiency prediction’ subsections for more information. Wang et al. (27) and Kim et al. (23) did not perform 10-fold cross validation, which is indicated as ‘NN’ - not applicable – in Table 1.

**Table 1.** Performance on benchmark datasets for on-target cleavage efficiency prediction

Dataset	Model	Metric	Value
DeepHF wildtype	(27), RNN	Hold-out SCC	0.8555
DeepHF wildtype	(27), RNN	10-fold CV SCC	NA
DeepHF wildtype	This study, C E	Hold-out SCC	0.8392
DeepHF wildtype	This study, C E	10-fold CV SCC	0.8066
DeepHF eSpCas9	(27), RNN	Hold-out SCC	0.8491
DeepHF eSpCas9	(27), RNN	10-fold CV SCC	NA
DeepHF eSpCas9	This study, R E	Hold-out SCC	0.8220
DeepHF eSpCas9	This study, R E	10-fold CV SCC	0.6927
DeepHF SpCas9-HF1	(27), RNN	Hold-out SCC	0.8512
DeepHF SpCas9-HF1	(27), RNN	10-fold CV SCC	NA
DeepHF SpCas9-HF1	This study, R E+M	Hold-out SCC	0.8364
DeepHF SpCas9-HF1	This study, R E+M	10-fold CV SCC	0.7900
geCRISPR V520	(26), mono binary	Hold-out PCC	0.6700
geCRISPR V520	(26), mono binary	10-fold CV PCC	0.6800
geCRISPR V520	This study, C E+M	Hold-out PCC	0.6055
geCRISPR T3619	This study, C E+M	10-fold CV PCC	0.5926
DeepCpf1 H1	(23)	Hold-out SCC	0.7600
DeepCpf1 H1	This study, R E	Hold-out SCC	0.7283
DeepCpf1 H2	(23)	Hold-out SCC	0.7400
DeepCpf1 H2	This study, C E+M	Hold-out SCC	0.7184
DeepCpf1 H3	(23)	Hold-out SCC	0.5800
DeepCpf1 H3	This study, R E	Hold-out SCC	0.5478
DeepCpf1 train	(23)	10-fold CV SCC	NA
DeepCpf1 train	This study, C E+M	10-fold CV SCC	0.5165

The performance of all trained models is given in Supplementary Table S3. Although in the original work on DeepHF datasets, the RNN-based models have been found to be superior to CNN-based ones (27), in our analysis, this distinction was not as pronounced. Some CNN-based GuideHOM models outperform RNN-based ones: for example, for the wildtype, the CNN-based model performs better. From the performance of the best models, it becomes clear that a combination of ELBO and MSE loss functions performs better than ELBO only. Let us consider the following use case: we would like to choose a model for gRNA selection for asCas12a editing experiment in human HEK293T cell line. We aim at maximizing the on-target efficiency but are not interested in minimizing the off-target effect. In Table 2, we provide a subset of performance measures for this case.

**Table 2.** Comparison of model predictions of the on-target cleavage efficiency for the AsCas12a subset

Model	ρ_68_	ρ_95_	ρ_99.7_	PCC	SCC	*r* ^2^
DeepCpf1 R E:H1	0.69	0.95	1.00	0.74	0.73	0.55
DeepCpf1 C E+M:H2	0.68	0.93	0.99	0.72	0.72	0.52
DeepCpf1 C E:H1	0.67	0.93	0.99	0.73	0.72	0.53
DeepCpf1 C E:H2	0.68	0.93	0.99	0.71	0.71	0.51
DeepCpf1 C E+M:H1	0.67	0.93	0.99	0.72	0.71	0.52
DeepCpf1 R E:H2	0.71	0.95	1.00	0.71	0.71	0.51
DeepCpf1 R E+M:H1	0.70	0.96	1.00	0.72	0.70	0.52
DeepCpf1 R E+M:H2	0.72	0.95	1.00	0.67	0.66	0.45
DeepCpf1 R E:H3	0.27	0.67	0.95	0.51	0.55	0.26
DeepCpf1 C E:H3	0.25	0.63	0.92	0.50	0.53	0.25
DeepCpf1 C E+M:H3	0.24	0.62	0.91	0.49	0.51	0.24
DeepCpf1 R E+M:H3	0.31	0.73	1.00	0.42	0.46	0.18
Cas12a pair E	0.59	0.87	0.96	0.57	0.57	0.32
Cas12a pair E+M	0.59	0.86	0.96	0.56	0.57	0.31

As can be seen from Table 2, we have a choice between models trained on DeepCpf1 and on Cas12a gRNA–target pair set. All DeepCpf1-based models show acceptable confidence intervals and good *r*^2^, Pearson and Spearman Correlation Coefficients.

For this test case, we are not interested in off-target effects, therefore DeepCpf1 R E is the model of choice (it outperforms all others on H1 and H3 datasets in Spearman Correlation Coefficient). We also can use all good models as an ensemble, which will result in an improvement of 1-2% in the correlations at the excess of increased uncertainty since we would have to sum the variances to get the correct uncertainty estimation.

Table 3 shows the results for the Jost et al. dataset, where the difference between E+M and E only is negligible. Compared with the model (25), changed accordingly to reflect the difference in the input size (we used as input 23 nt sequence, including the gRNA spacer and PAM, whereas in the Jost *et al.* (25) analysis, two upstream and one downstream flanks were also included, resulting in the input size of 26 nt), the performance improves from 0.617 to 0.625. For the purpose of the comparison, we modify the original code from the supplementary file (25) by taking the sequence parts from second to 24-th nucleotide and changing the input size to (4, 23, 2) instead of (4, 26, 2). A 2D visualization of GuideHOM representations allows for intuitive design for gene editing and modulation of gene expression experiments. The representation computed by the Hit-or-Miss layer can be interpreted as either coordinates of a gRNA in the space of all possible gRNAs or a gRNA/target pair in the space of all possible gRNA/target pairs depending on the model (the former is for RNN and 1D-CNN models, and the latter is for 2D-CNN models used for the Cas9 and Cas12a gRNA–target pair sets). Due to the gradient descent optimization, the sequences in such a space are arranged according to sequences and cleavage efficiencies (similar sequences are expected to occupy a compact subspace of the guide space). The dimensionality of such a guide space is determined by the number and output dimensionality of the HOM capsule layers.

**Table 3.** Comparison of the results for the subset from (25) study

Metric	Jost *et al.* E	Jost *et al.* E+M
ρ_68_	0.7222	0.7125
ρ_95_	0.9208	0.9155
ρ_99.7_	0.9838	0.9828
PCC	0.7849	0.7805
SCC	0.7003	0.6982
*r* ^2^	0.6161	0.6192

**Figure 4.**
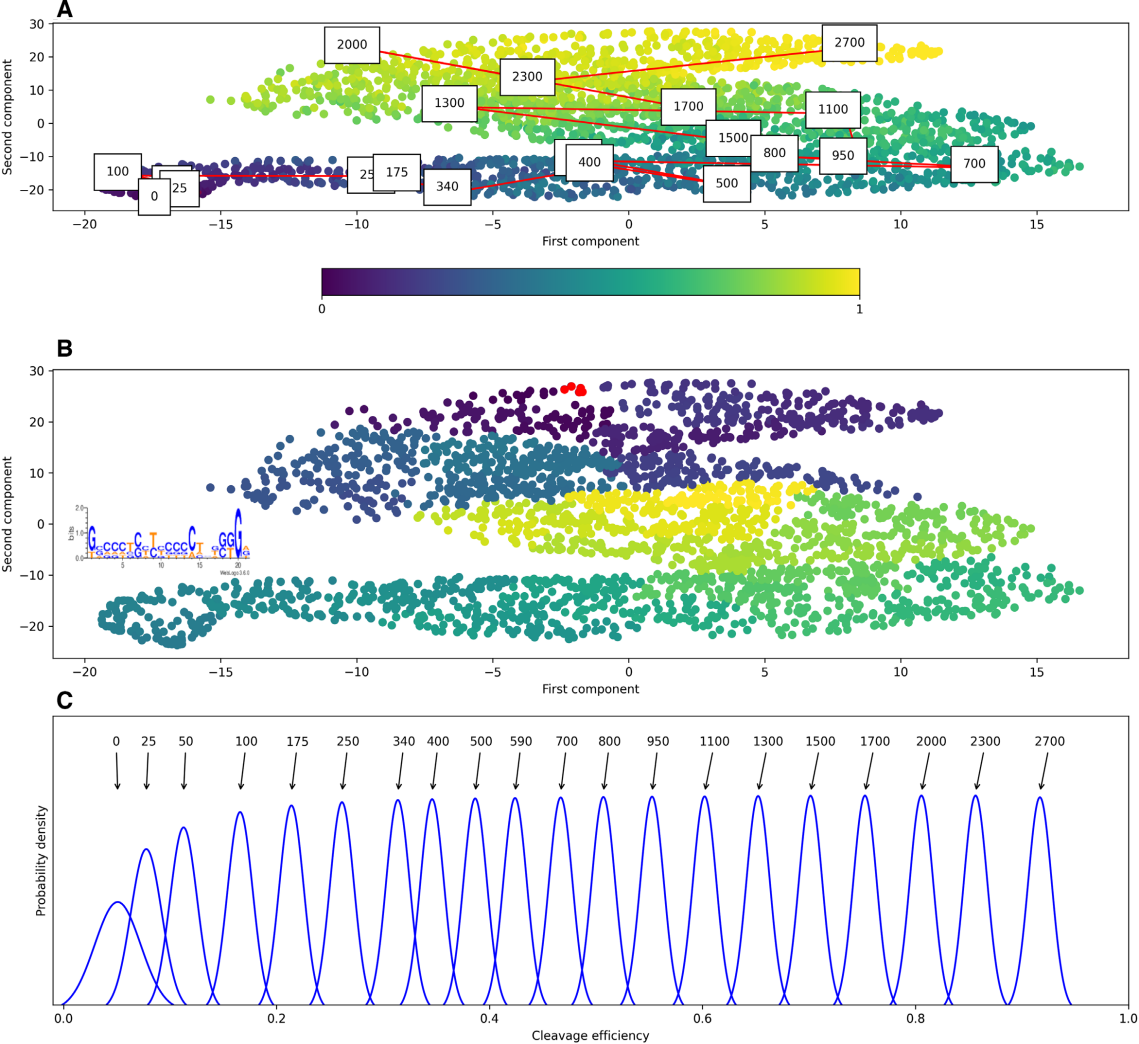
2D visualization of the guide space with NCVis. The active and inactive gRNAs scatter around the plane according to their mean cleavage efficiency (**A**) and cluster into small subgroups based on the sequence determinants of cleavage efficiency (**B**). The color gradient at (A) denotes mean efficiency (yellow – the largest, purple—the smallest). Color of the dots denotes the cluster label at (B). One of the groups is shown as red dots and the sequence logo of that cluster is shown in the inset of (B). A path along the gradient of color (red lines in panel (A) gives a functional set of gRNAs sufficient for modulation of gene expression (**C**). The numbers at the plot (A) and at the plot (B) denote the same gRNAs. The gRNA sequences are shown in the Supplementary Table S4. For this figure, the DeepHF Cas9 C E wildtype model was used.

**Figure 5.**
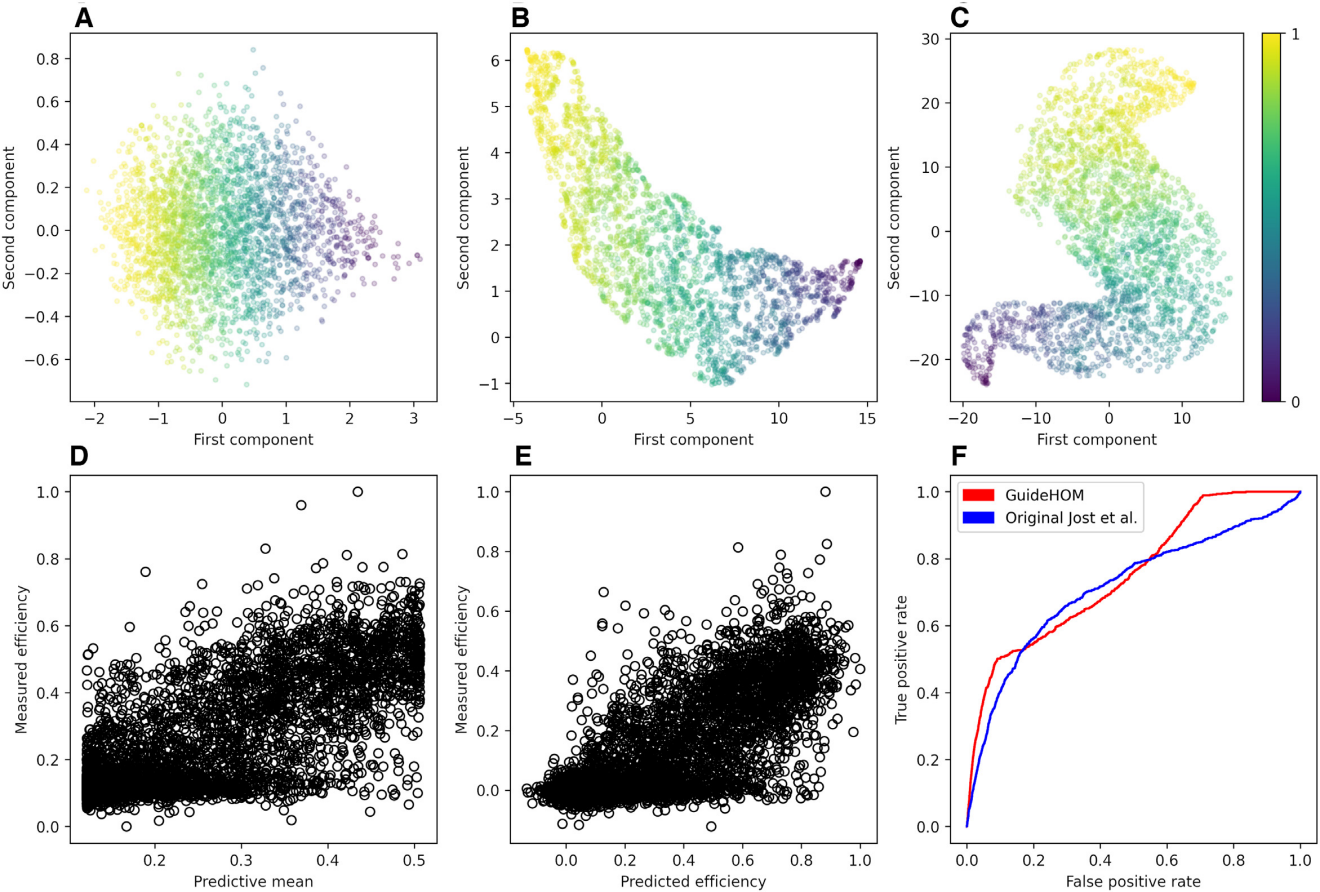
2D visualization of the guide space with different visualization methods: (**A**) PCA, (**B**) UMAP, (**C**) NCVis. All views illustrate the same properties. Predictions vs observed activities for (**D**) GuideHOM and (**E**) Jost et al. models. (**F**) ROC curves of the GuideHOM and the Jost *et al.* model (25) on the dataset of Peng *et al.* (42). For this figure, the DeepHF Cas9 C E wildtype model was used.

2D visualization of the guide space provides actionable insights into the variety of functional gRNAs available for a gene of interest. Figure 4 presents an example of the guide space for LOC440792 gene. The representation of the guide space (Figure 4A) as a scatter plot with mean cleavage efficiency denoted as the point color is more visually appealing and intuitive than spreadsheets that are commonly used for the same purpose. On the scatter plot, the most efficient sequences are clustered together with sub clusters formed according to different sequence determinants of cleavage efficiency (Figure 4B). An example of a sequence motif associated with one such cluster is shown on Figure 4B. The sequence determinants are specific for each cluster, which implies no simple linear dependency of cleavage efficiency and/or the cleavage efficiency variance on the sequence motifs. While efficient gRNAs share some common features, such as high GC content, there is no single unifying motif. Instead, any nucleotide change increases, decreases or, in some cases, does not perceptibly affect the efficiency. Visual arrangement allows for quick search for most efficient gRNA or a functional gRNA sets. Different functional sets of gRNAs can be found by following the color gradient from the most efficient to the least efficient (from purple to yellow dots in Figure 4 A). An example of this path is shown in Figure 4A (numbers in boxes denote the number of gRNAs in the gRNA set, the same numbers correspond to cleavage efficiency distributions on Figure 4C). The set of gRNAs from Figure 4C found by following the color gradient of the gRNA space visualization, that has the required properties—gRNAs with all range of cleavage efficiency values, can be used to study the metabolic pathway responsible for hyperprolinemia (since the LOC440792 is associated with it, https://www.genecards.org/cgi-bin/carddisp.pl?gene=LOC440792) by performing *in vitro* experiments with each of the gRNAs and measuring the levels of proline. All sequences from this set are presented in the Supplementary Table S4. All logo sequences for the identified clusters are presented in Zenodo repository. The clustering and cleavage efficiency gradients in the 2D visualization of the guide space are apparently agnostic to the method of visualization. We experimented with PCA (Figure 5A), UMAP (arXiv preprint arXiv:1802.03426, 2018) (Figure 5B) and NCVis (41) (Figure 5C), and the results are qualitatively the same: each visualization method yields partitioning of the gRNAs into motif-dependent subclusters, and the color gradient is sufficient for delineating a functional set of gRNAs.

For all visualizations in Figures 4 and 5 (except for D and E where we use test set of Cas9 off-target dataset (25)), we use the LOC440792 gene (2781 gRNAs) and the GuideHOM DeepHF wildtype C E+M model. The visualizations are not supervised with respect to either the average cleavage efficiency or the standard deviation, therefore representations encoded in the outputs of capsule network are enough to produce the scattering of gRNAs on the plot by color, which shows once again that the model has learned the representations related to cleavage efficiency.

In addition to reproducing the training routines from previous studies, we conducted a 10-fold cross-validation analysis. Most of the previous studies we based our work on did not perform 10-fold cross validation, so that we cannot compare our performance with that reported in these studies without reproducing them in their entirety, which is outside of the scope of this work. The cross validation results are available in Supplementary Table S7. Under the 10-fold cross validation scenario, the values of quality metrics tend to be smaller than in the hold-out dataset case by about 0.05 (e.g. DeepHF wildtype CNN ELBO reaches 0.8392 for the hold-out and 0.8066 on average in 10-fold CV, with standard deviation of 0.0216). The largest gap was observed for the Cas12a off-target model, which, in the10-fold CV, on average, does not yield acceptable confidence intervals with respect to *p*_68_, *p*_95_ and *p*_99.7_. This could be explained by the small size of the dataset, which only includes 1597 gRNA-target pairs, less than half of the next smallest dataset, geCRISPR, with 3619 gRNAs. For the rest of the models, the confidence intervals, on average, remain acceptable. There is also a substantial gap in the performance between the RNN and CNN-based models for the geCRISPR dataset, with RNN ELBO and RNN ELBO+MSE performing worse than the CNN counterparts (Pearson correlation 0.3882 and 0.4403 versus 0.5946 and 0.5926). The remaining models do not yield significant differences in performance either between RNN and CNN or between ELBO and ELBO+MSE. Overall, the cross validation study shows that the GuideHOM architecture is robust to overfitting provided there is enough data to train it on.

### GuideHOM solves off-target cleavage regression with acceptable confidence intervals

Our approach allows not only for the prediction of the on-target cleavage efficiency based on a single gRNA but also, with minimal changes to the architecture (see ‘Materials and Methods’), for the prediction of off-target cleavage efficiency from a pair of a gRNA and the target. For the Cas9 gRNA–target pair dataset (25), we train the model on 80% of the training set and test it on the remaining 20%. We found an *r*^2^ value of 0.625, as compared with 0.617 in the original study, and an acceptable confidence interval (0.7176 on Ymean + 1, 0.9124 on Ymean + 2, 0.9804 on Ymean + 3). The prediction plots for the test set are shown in Figure 5D and E.

The original Cas9 gRNA-target pair model (25) is obtained by slightly modifying the supplementary file in Jupyter Notebook. The sgRNA and genome target sequences are trimmed to remove the first two and one last nucleotides (the flanks) in order to leave only the gRNA and the PAM. The parameter input_shape in the model definition is changed to (4, 23, 2).

The model trained on the Cas9 gRNA-target pair dataset provides Cas9 cleavage efficiency estimates for guide and target pairs with small numbers of mismatches between the guide and the target. This information can be used to solve the off-target classification task using the Peng *et al.* (42) classification dataset to test our models. We compared the performance of our models with that of the reproduced Jost et al. model and obtain a better AUROC of 0.7528 (the Jost *et al.* model scored 0.6777). The ROC curves for these models are shown in Figure 5F. For the test, we removed from the Peng et al. dataset all pairs that have more than 6 mismatches to reduce the number of negatives. According to the ROC curves, our model tends to produce fewer false positives for low decision thresholds.

We next trained a regression model for Cas12a gRNA-target pair dataset. We split the dataset of (28) into 90% for training and 10% for testing. We obtain Spearman Correlation Coefficient of 0.6 and a borderline acceptable confidence interval (0.58 on σ, 0.86 on 2σ, 0.95 on 3σ). The variability and lower correlation are likely due to the small dataset size of only 1565 gRNA-target pairs. We did not use the PAM information because all sequences in the dataset contained the TTTA PAM but use additional flank region so the overall input length is 23. The limitations of the Cas12a gRNA-target pair dataset are also reflected in the corresponding learning curves (Figure 2A and B). The learning curve and mean standard deviations converge towards 0.6 and 0.2, respectively, and are comparable to the results obtained with other small samples, such as the DeepCpf1 set, but the correlation (Spearman correlation coefficient) is weaker than that for DeepHF. To our knowledge, this is the first attempt at off-target cleavage efficiency regression for asCas12a and the performance of the model is promising.

### Diversity of CRISPR off-target effect predictions demonstrated by analysis of gRNA-target pairs

We analyze an empirically validated sgRNA library for 2400 genes that are essential for robust cell growth (25). The consistency and slight but significant superiority of results obtained with our method compared to those in the original study (*r*^2^ value of 0.625 versus 0.617 for the Jost et al. model, see details above) supports the utility of our approach. As an example of a practical application, we provide the top 5 gRNAs with mean and variance values for each gene in the Homo sapiens reference genome (hg38) chromosome 22. An example of the output for gene SERPIND1 is shown in Table 4. We suppose that the gRNAs could be used right away to plan the CRISPR/Cas9 gene editing experiment with the gene SERPIND1.

**Table 4.** Example of top 5 gRNAs for Cas9. The model is DeepHF WT R E

Start	Sequence	Strand	Mean	Variance
5273	GGATCAGCTAGAGAAAGGAGGGG	+	0.9344	0.0122
12819	CAGCGGCATGAACCCCACCGTGG	-	0.9310	0.0120
10683	TCATGGCAGAAAGAATGGAGAGG	+	0.9230	0.0119
7403	GTGTGTGGACAGATCAGGAGGGG	-	0.9264	0.0119
4807	AGAGACAAAGTTCCCACCAGGGG	-	0.9260	0.0119

Cas-OFFinder (39) was used to make a list of potential targets. A detailed description of the pipeline used for human genome analysis is presented in ‘Materials and Methods’. To apply our approach for prediction of mismatched gRNA cleavage efficiency and search of sgRNAs with systematically modulated activities, we analyze 1000 random gRNAs which are extracted from the top 10 highly efficient gRNAs (see Supplementary Table S5 for the 1000 extracted). For each of these gRNAs, all possible off-targets with no more than 6 mismatches are selected from human chromosome 22. The mean cleavage efficiency and cleavage efficiency variance are computed using the GuideHOM model trained on the Cas9 gRNA–target dataset (CNN ELBO) for each identified off-target gRNA–target pair.

**Figure 6.**
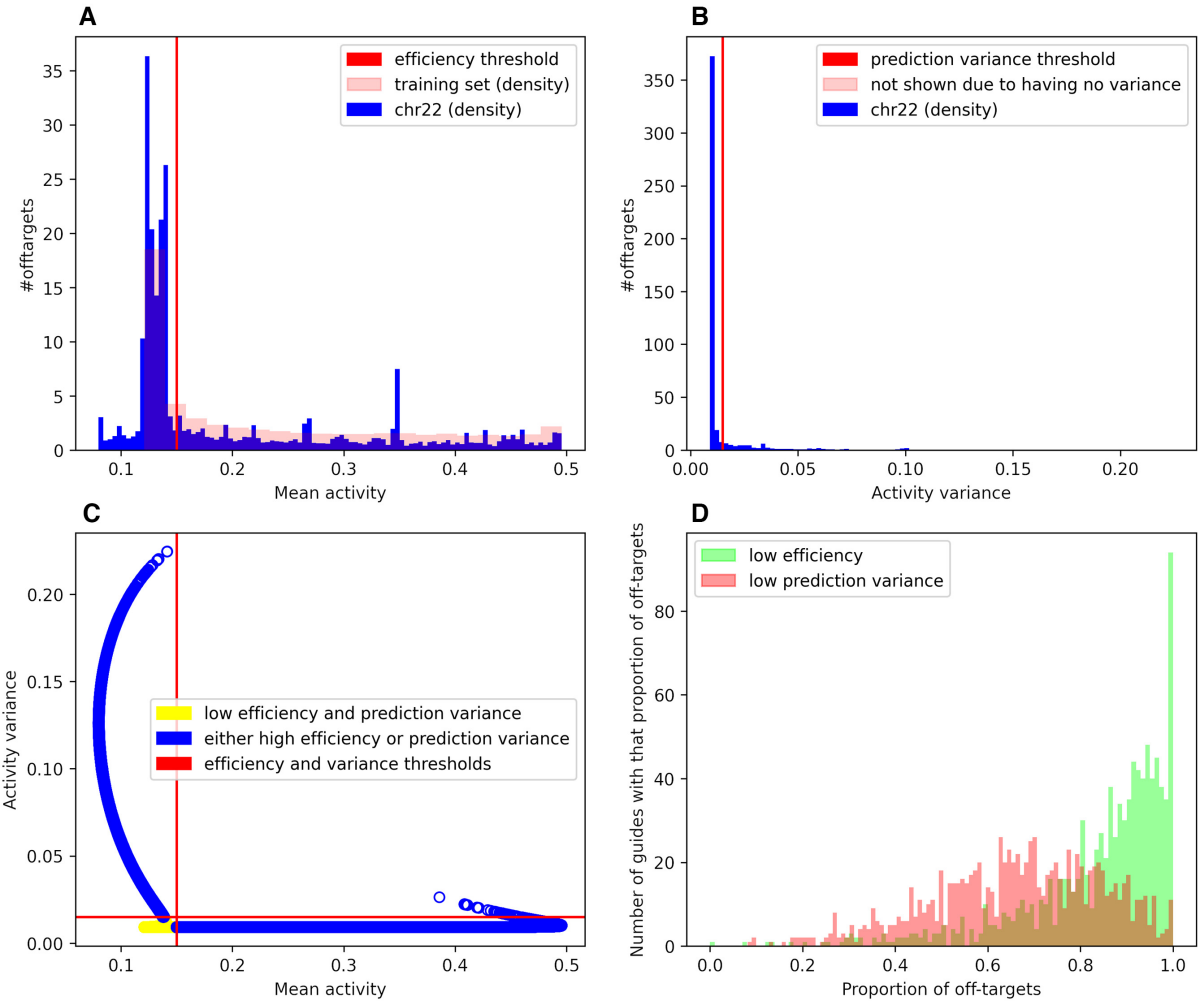
Analysis of off-targets in Chromosome 22 shows the existence of different off-target categories: (**A**) distribution of mean off-target cleavage efficiency for real known values extracted from the training set (pink) and for predictions for Chromosome 22 (blue) with cleavage efficiency threshold of 0.15. (**B**) distribution of off-target cleavage efficiency variance for Chromosome 22 with prediction variance threshold of 0.015. (**C**) Mean versus Variance plot for off-targets from gRNAs extracted from top 10 of different Chromosome 22 genes with indicated cleavage efficiency and prediction variance thresholds. (**D**) Distributions for proportions of off-targets with low efficiency (green) and off-targets with low prediction variance (pink) for 1000 of top10 gRNAs.

The inclusion of uncertainty estimates in the off-target analysis provides for two orthogonal axes that characterize off-target properties: cleavage efficiency and stability of prediction, where cleavage efficiency shows how probable is the cleavage of the DNA strand with this particular target and this particular gRNA, and prediction variance shows how robust the prediction of cleavage efficiency is. Figure 6A shows that most of the predicted off-targets (74%) have low cleavage efficiency (<0.15) but there is a minority of highly efficient off-targets (26%) that should be avoided for any application that depends on the minimization of off-target effects. Most off-targets (82%) have small variance (<0.015, see Figure 6B, they differ from the predicted mean cleavage efficiency only by 0.1223 at worst, with the probability of 0.65), so there is a negligible chance that these off-targets are incorrectly predicted. However, a minority of the off-targets with large variance (18%) should be removed from experimental validation because gRNAs with such cleavage efficiency variance on off-targets can be problematic in the experiments (the real cleavage efficiencies of these gRNAs can differ from the predicted mean so much that one can not be sure in the quality of the cleavage efficiency prediction even with the confidence intervals taken into account).

Most off-targets with cleavage efficiency variance <0.015 and mean cleavage efficiency <0.15 are located in the left bottom corner of the plot on Figure 6C. For gene editing, only the gRNAs with the smallest number of off-targets with high prediction variance and the highest on-target cleavage efficiency should be selected. For gene expression modulation, a set of gRNAs that produce off-targets with chosen levels of prediction variance can be used. In this case, the low variance of the off-target effect allows the selection of a suitable set of gRNAs with predictable results. For each of 1000 randomly selected highly efficient gRNA, the proportions of the different kinds of off-targets are shown in the Supplementary Table S6. Figure 6D shows that most gRNAs have largely off-targets with both low cleavage efficiency and low prediction variance (the mean fraction of off-targets with low cleavage efficiency is 0.80, with the standard deviation of 0.13, and the mean fraction of off-targets with low prediction variance was 0.62, with the standard deviation of 0.17). However, for some gRNAs, many off-targets with high cleavage efficiency and high prediction variance were identified (on average, the proportion of both off-targets with high cleavage efficiency and low prediction variance and off-targets with low cleavage efficiency and high prediction variance is 58% with 14% standard deviation, and there is an insignificant amount of the off-targets that both have high cleavage efficiency and high prediction variance, only 23 gRNA out of 1000 have them, there are only 56 such off-targets out of 1 994 178 total. Such properties would exclude the gRNAs from the candidate pool for an experiment. The proportion of off-targets with low cleavage efficiency and low prediction variance can be used to select gRNA for different types of experiments because the off-targets with low cleavage efficiency and low prediction variance are distributed differently for different gRNAs. Thus, the results show that our approach is useful for the prediction of mismatched gRNA cleavage efficiency and evaluation of systematically attenuated gRNAs that can be used to control gene expression, from tuning biochemical pathways to identifying suppressors for diseases and stress conditions.

### Neural network interpretation demonstrates the sequential preferences for on-target and off-target cleavage

**Figure 7.**
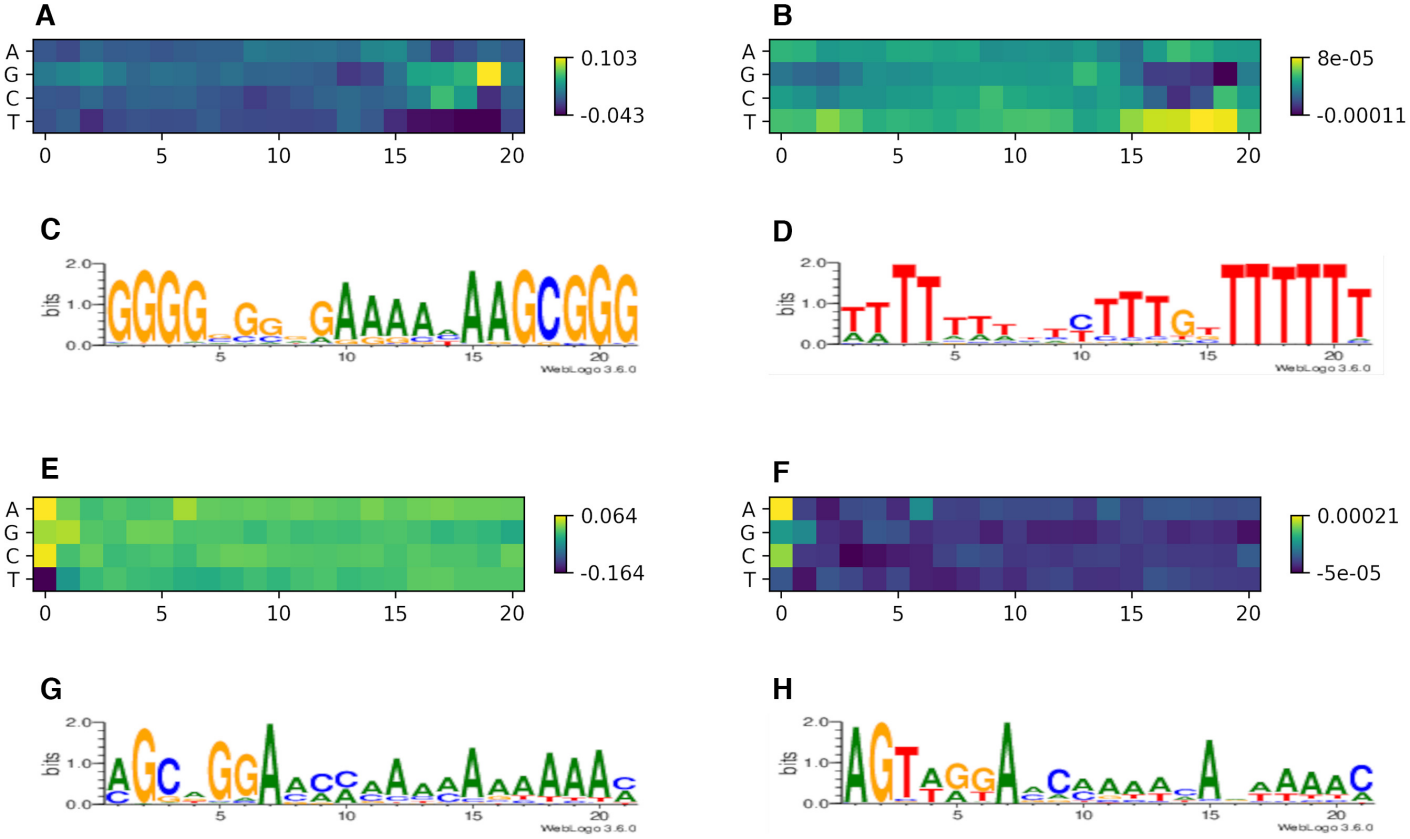
Neural Network Interpretation for Cas9 and Cas12a on-target models shows the importance of seed region which is located at the right hand side for Cas9 (**A**) and (**C**), and at the left hand side (**E**) and (**G**) for the prediction of cleavage efficiency. The same importance is observed for the variance of predictions (**B**) and (**D**) for Cas9, (**F**) and (**H**) for Cas12a). The models used are DeepHF WT C E and DeepCpf1 C E.

We use the Accumulated Local Effects (ALE (37)) to explain predictions of trained models. A single ALE value shows the influence of a feature towards the output of the network. In case of one-hot encoded features, it shows the impact of presence and absence of a feature. ALE is a black-box explanation method that requires very few assumptions about the model, so it is perfect for our case, with model that predicts not only the cleavage efficiency, but also its variance. We compute the influences for all nucleotides towards mean efficiency and variance, plot the heatmaps and logo sequences of the resulting matrices. The logo sequences and heatmaps show the preferences for gRNA sequence in on-target cleavage (Figure 7) and gRNA-target pair in off-target cleavage (Supplementary Figure S1 for Cas9 and Supplementary Figure S2 for Cas12a). The region of about 4–5 nt located near the PAM is more important for the prediction than the rest of the sequence. This holds for both Cas9 and Cas12a—for Cas9 it is located on the 3′ end, and for Cas12a it is located on 5′ end. The mechanics of PAM and target recognition implies the importance of the seed region. The recognition of target starts from PAM and goes towards the end of the target through the seed region. If there is a mismatch in the seed region, the cleavage is highly unlikely. Our model captures the importance of seed region without any additional supervision from the user. We didn’t indicate it in any way (a possible way to indicate would be to give the network a mask in addition to the sequence, which we didn’t do), the model had to learn it on its own to use in cleavage efficiency prediction.

Importance heatmaps for efficiency and variance tend to mirror each other, not exactly, but very close. For example, in Cas9 (Figure 7A and B), the Gs and Cs in the seed region are very important for efficiency, but for variance, Ts and As are more important. Basically, if a gRNA has Cs and Gs in the seed region, the variance will be low, but the cleavage efficiency will be rather high. However, this holds poorly for Cas12a. In Figure 7E and F, in two first letters of the seed, both variance and efficiency matrices value As and Gs, and undervalues Ts. The rest of the seed and the rest of the sequence after the seed have the same overall importance. For mismatching gRNA-target pairs (Supplementary Figure S1) in Cas9, the mismatches in PAM are very important, as important as in the seed region. The lack of importance for Ts in the seed region for Cas12a is also reproduced in Cas12a off-target model despite using different datasets (Supplementary Figure S2). The same holds for Gs and Ts in seed region of Cas9 (Supplementary Figure S1). It shows that models learn the same features from different datasets of the same Cas effector – those features are not dataset-specific, but Cas effector-specific.

## DISCUSSION

In this work, we introduce Deep Kernel Learning-based methods for estimation of confidence intervals for gRNA-target cleavage efficiency. Using a number of publicly available datasets, we construct a set of effector- and cell line-specific models. Our results indicate that deep kernel learning offers substantial advantages over basic deep learning in the estimation of the efficiency of CRISPR-mediated target cleavage. The methods we develop here are distinct from all previously described ones in that an uncertainty estimation is explicitly given. The uncertainty of the model provides valuable clues for the design of gene editing experiments, allowing the end user to select not only gRNAs that are optimal with respect to the on-target and off-target efficiency, but also those that are the most predictable in their behavior in experiments. For example, a researcher probably should pick a low variance gRNA with a lower predicted efficiency compared to a gRNA with a higher efficiency but a wider confidence interval. The confidence interval is important in two cases: when there are many gRNAs to select from and when highly specific editing is required. Given that experimental researchers routinely use at least 10 different gRNAs to target the gene of interest, the confidence intervals become a valuable tool, because they allow the likelihood of an experimental failure to be minimized through optimal selection of gRNAs. As for the specific editing, there are many tasks that require not only silencing a gene, but silencing it using a specific region of the gene as the target.

A salient example is analysis of small RNAs, including miRNAs and their targets. In cases when there are many predicted target sites for a small RNA (each can be as short as 8 bp), inducible Cas9 experiments and multiplexed single guide RNAs can be used to generate hundreds of targeted mutations in parallel (43–45). When the goal is to target different regulatory sites in many genes, as in the case of miRNA targets, and analyze Cas9-induced mutations, it seems to be advantageous to choose not the most efficient gRNA for a given gene, but those gRNAs that match the sequences closest to the putative target sites of small RNAs, even such gRNAs are inferior in efficiency. The choice among the low efficiency gRNAs requires taking into account the variability in efficiency prediction, in order to reduce the uncertainty of the experimental results.

As an encoder, we used the Hit-Or-Miss capsule network, which is a capsule network without routing. This design is faster than traditional capsule networks because it omits the most time-consuming step, while maintaining nearly identical performance. We had to modify the setup for the HOM networks because they were initially designed for classification. In the classification setting, the representation learned by HOM capsules represents the difference from the template, that is, a perfect example of the class. In our setting, the HOM capsules learn a coordinate in the space of all possible gRNAs or gRNA-target pairs. The space is bounded by the sequence and cleavage efficiency. The two-dimensional visualization of this space is a natural way to present the gRNAs for the gene of interest. This visualization provides an overview of the gene targeting by gRNAs and helps in the selection of most efficient gRNAs or a set of gRNAs that are useful for gene expression modulation. The functional gRNA set can be formed by selecting the guides alongside the efficiency gradient that is shown by colors in the plots. Taking into account that the Hit-Or-Miss capsule network approach has been successfully applied for prediction of targeting efficiency for gRNA–target pairs, we use a recently published large-scale sgRNA mismatch dataset (25) for exploration of the ground rules of mismatched gRNA–target interactions. These data enable prediction of sgRNAs with intermediate cleavage efficiency against complete sets of expressed genes and demonstrate that the activities of mismatch-containing sgRNAs are determined by a variety of factors that can be captured using supervised machine learning approaches. The utility of the Hit-Or-Miss capsule networks for the study of complex global and local dependencies on spatially ordered functions, such as nucleotide sequences, including factors that regulate CRISPR gRNA cleavage efficiency, suggest that this approach will enable further characterization of the features of mismatched sgRNAs that contribute the most to their efficiency. Such features can be exploited for analysis of expression-phenotype relationships in mammalian cells. In a recent study, systematically attenuated sgRNAs for staging cells along a continuum of expression levels were employed for exploration of fundamental biological questions, specifically, for the analysis of gene-specific expression–phenotype relationships and expression-level-dependent cell responses at single-cell resolution (25). Our approach enables further investigation of these fundamental biological problems and could be useful for systematic large-scale studies of activities of individual genes in basic cell biology, drug development and functional genomics. Our work also shows that experimental design benefits from the estimation of the model uncertainty, which is provided by the Gaussian Process. The resulting models can be used for the design of Cas9/Cas12a-based gene editing experiments that maximize on-target efficiency and minimize the off-target effects, by taking into account the information on dataset-specific and replication-specific noise, and proportions of different off-target types. For example, when facing a choice between a gRNA that has a high number of inefficient and low variance off-targets and a gRNA with a small number of efficient but high variance off-targets, the experimenter should pick the former because the latter comprises a much greater uncertainty. The high-efficiency and high-variance off-targets are rare, but it is important to exclude the gRNAs that are associated with such off-targets from the set of selected gRNAs because including these can have unpredictable consequences for the experiment outcome. The proportions of off-targets with high and low efficiency, as well as high and low variance thus serve as additional metric to the number of off-targets, which is customarily used in experimental design, providing information that is lacking in most available tools. Compared to the performance of the point estimate models (DeepCpf1 (23), DeepHF (27), Jost *et al.* (25) etc), our model provides acceptable confidence intervals and comparable performance. There seems to be a trade-off between the uncertainty estimation and point estimation quality. This trade-off has been observed previously in a similar setting (40). For mere experiment design, the knowledge of cleavage efficiency (and its variance) is most of the time sufficient. However, neural networks are more than simply a way to estimate probabilities, they also can be used as black-box models for the biological process of interest. Such models allow the researcher to test hypotheses on biological processes *in silico*. To assess the compatibility of our models with the known features of the Cas protein-induced cleavage, we explained the predictions with Accumulated Local Effects and found that our model reproduced the known behavior of the system, namely, the dependence of cleavage on the seed region of the gRNA. This behavior was reproduced in both Cas9 and Cas12a models showing that our Capsule Network-Gaussian Process hybrid indeed builds the representations relevant for prediction of properties of CRISPR systems and is unlikely to result from overfitting.

## DATA AVAILABILITY

The code for reproduction of the models, tables and figures, can be found in the following repository: https://github.com/bakirillov/uace. The gRNAs with predicted cleavage efficiencies and variances can be found on Zenodo (https://doi.org/10.5281/zenodo.4773404) along with the logo sequences for gRNA clusters from LOC440792 gene. Demonstration of all models is available on Google Colab (ID: 1VJkhQeqW1OkMG0VJoxcpML7LtnkvTX9e).

## Supplementary Material

gkab1065_Supplemental_Files
