# Supplementary material for: Uncertainty-aware and interpretable evaluation of Cas9–gRNA and Cas12a–gRNA specificity for fully matched and partially mismatched targets with Deep Kernel Learning
Source: Nucleic Acids Res. 2021 Nov 17;50(2):e11. doi: 10.1093/nar/gkab1065 (PMC8789050; doi:10.1093/nar/gkab1065)
Supplement: gkab1065_Supplemental_Files [file gkab1065_supplemental_files.zip › Supplementary Figures.pdf]

Supplementary Figure 1: Heatmaps and logo sequences of feature importance for Cas9 off-target efficiency.

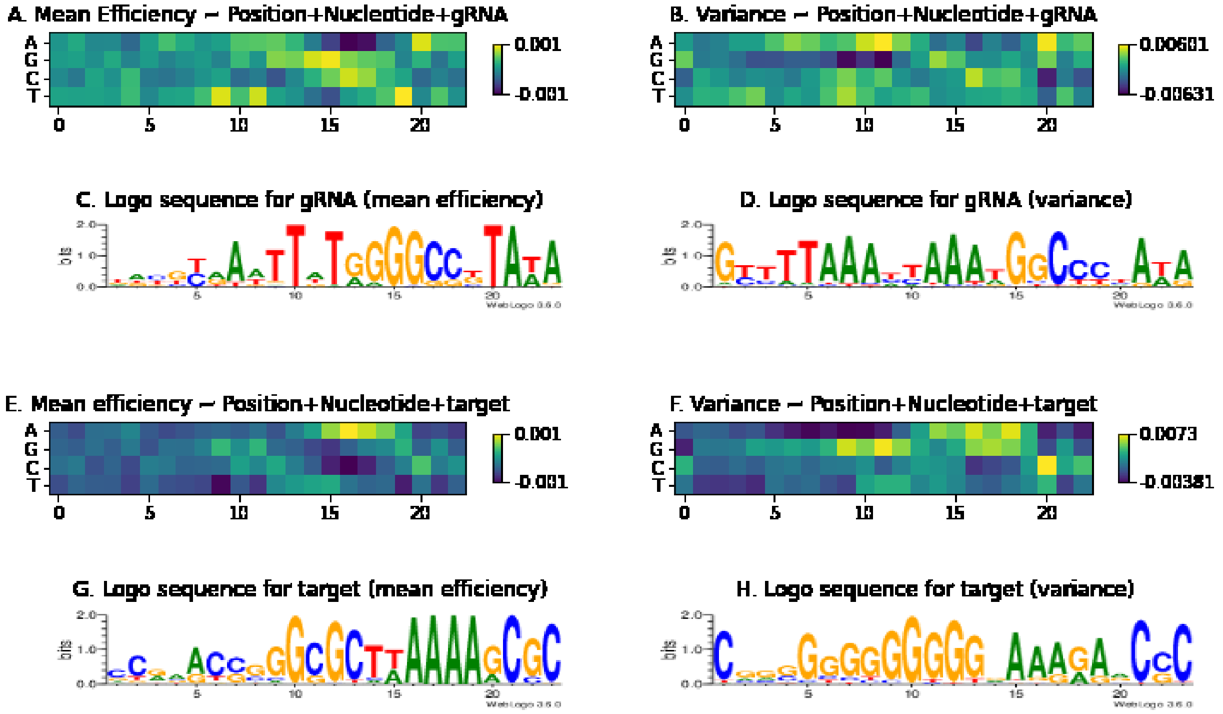

The model is trained to minimize ELBO. The same importance of seed and PAM as in Figure 7A,B,C,D is observed on heatmaps, but logo sequences highlight additional dependency along the whole gRNA sequence.

Supplementary Figure 2: Heatmaps and logo sequences of feature importance for Cas12 off-target efficiency.

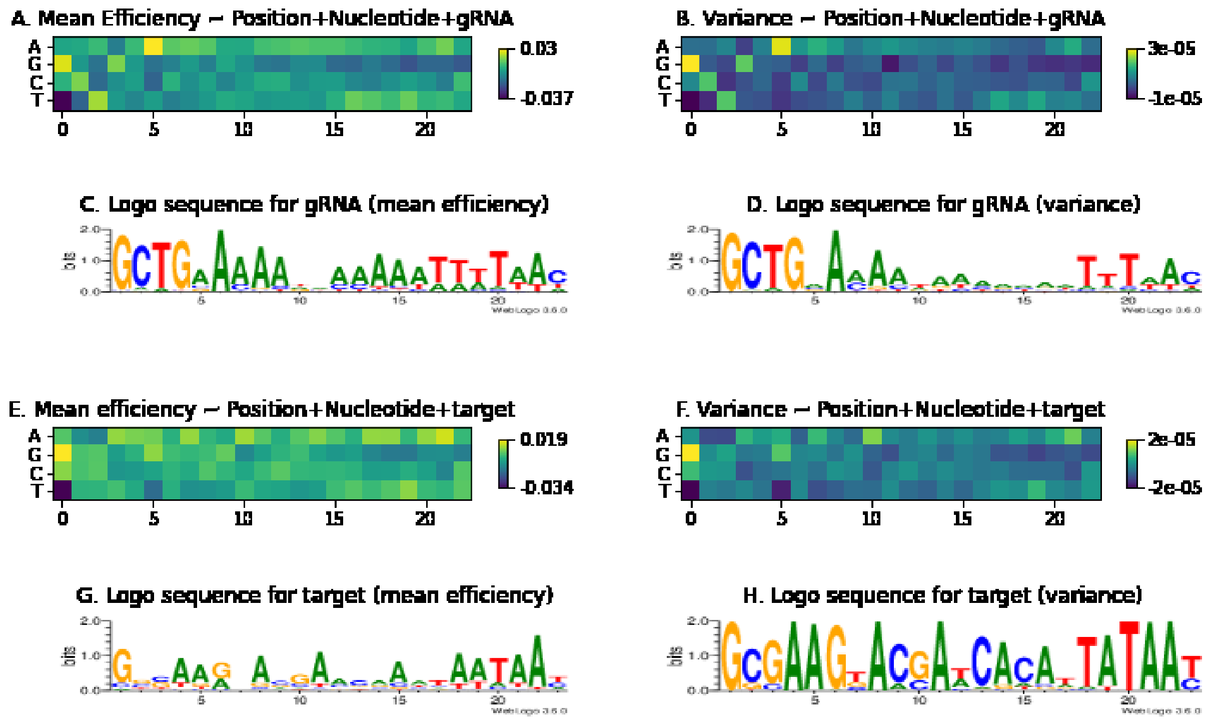

The model is trained to minimize ELBO. The same importance of seed and PAM as in Figure 7E,F,G,H is observed on heatmaps (reversed compared to Cas9 plots), but logo sequences highlight additional dependency along the whole gRNA sequence, as well as in case of Supplementary Figure 1.
