# Supplementary material for: Uncertainty-aware and interpretable evaluation of Cas9–gRNA and Cas12a–gRNA specificity for fully matched and partially mismatched targets with Deep Kernel Learning
Source: Nucleic Acids Res. 2021 Nov 17;50(2):e11. doi: 10.1093/nar/gkab1065 (PMC8789050; doi:10.1093/nar/gkab1065)
Supplement: gkab1065_Supplemental_Files [file gkab1065_supplemental_files.zip › SupplementaryFile2.pdf]

Supplementary Information

Uncertainty-aware and interpretable evaluation of Cas9-gRNA and Cas12a-gRNA specificity for fully matched and partially mismatched targets with Deep Kernel Learning

Bogdan Kirillov[1,2], Ekaterina Savitskaya[1], Maxim Panov[3], Aleksey Y. Ogurtsov[4], Svetlana A. Shabalina[4], Eugene V. Koonin[4], and Konstantin V. Severinov[1,2,5,6*]

1 Center for Life Sciences, Skolkovo Institute of Science and Technology, Moscow 143026, Russia. 2 Center for Precision Genome Editing and Genetic Technologies for Biomedicine, Institute of Gene Biology, Russian Academy of Sciences, Moscow 119334, Russia. 3 Center for Computational and Data-Intensive Science and Engineering, Skolkovo Institute of Science and Technology, Moscow 143026, Russia. 4 National Center for Biotechnology Information, National Library of Medicine, National Institutes of Health, Bethesda, MD 20894, USA. 5 Institute of Molecular Genetics, Russian Academy of Sciences, Moscow 123182, Russia. 6 Waksman Institute for Microbiology, Rutgers, The State University of New Jersey, Piscataway, NJ 08854, USA.

* Corresponding author

Konstantin V. Severinov, email: severik@waksman.rutgers.edu

Legends and captions for supplementary tables.

Supplementary Table 1 - Model definitions.

This supplementary table contains descriptions of different GuideHOM architectures used in the study - 2D-CNN, RNN and CNN.

The parameters are as follows:

1. capsules - the number of primary capsules;
2. output vector size - the dimension of vector with capsule outputs;
3. channels - the number of channels in primary capsules;
4. output classes - the number of class capsules;
5. guide and target lengths - lengths of gRNA and target;
6. CoordConv1d filters, stride - number of CoordConv1d convolutions and stride;
7. preprocessing activations - activations in preprocessing layers;
8. batch size - number of examples in a batch for training;
9. LSTM layers - number of LSTM layers for RNN-based architecture;
10. hidden units - number of hidden units in one LSTM layer (for RNN-based architecture);

Supplementary Table 2: Used train-test splits

This supplementary table contains information about train-test splits that each published previous study used. The columns are as follows:

1. Dataset - the name of the study;
2. Train - the proportion of data assigned for training;
3. Test - the proportion of data assigned for testing;
4. Validation - the proportion of data assigned for validation;
5. Cell line/species - the species and cell lines that each previous study used.

Supplementary Table 3: Performance of the models

This supplementary table contains information about predictive performance of the trained models. The columns are as follows:

1. Index column - name of the model which consists of dataset name, used architecture (C for CNN, R for RNN) and loss function (E for ELBO, E+M for ELBO+MSE);
2. 0.68, 0.95, 0.997 - values of p_{68}, p_{95} and $p_{99.7}$;
3. PCC and PCC-pval - Pearson Correlation Coefficient with respective p-value;
4. SCC and SCC-pval - Spearman Correlation Coefficient with respective p-value;
5. rsquared - values of r^2.

Supplementary Table 4: gRNAs found in LOC440792 gene

This supplementary table contains results for gRNAs found in LOC440792 gene that were used to construct Figure 5. The columns are as follows:

1. Index column - number of gRNA;
2. gRNA - the sequence of gRNA;
3. strand - the strand where gRNA is located (+ for forward, - for reverse);
4. X and Y - the PCA coordinates of the gRNA in the gRNA space;
5. cluster - the cluster label assigned to the gRNA.

Supplementary Table 5: 1000 randomly extracted highly efficient gRNAs

This supplementary table contains results for gRNAs found in Chromosome 22 of HG38 that were used to construct Figure 6. The columns are as follows:

1. Index column - number of gRNA;
2. gRNA - the sequence of gRNA;
3. strand - the strand where gRNA is located (+ for forward, - for reverse);
4. mean - the mean cleavage efficiency of the gRNA;
5. variance - the predicted variance of cleavage efficiency for the gRNA.

Supplementary Table 6: Proportions of different off-targets for 1000 randomly extracted highly efficient gRNAs

This supplementary table contains results of off-target analysis for gRNAs found in Chromosome 22 of HG38 that were used to construct Figure 6. The columns are as follows:

1. Index column - the number of gRNA, corresponds to the same column of Supplementary Table 5;
2. total - the total number of off-targets for gRNA;
3. low efficiency - the proportion of off-targets with efficiency lower than the efficiency threshold of 0.15;
4. low variance - the proportion of off-targets with variance lower than the variance threshold of 0.015;
5. low efficiency and variance - the proportion of off-targets that have both low efficiency and low variance;
6. high efficiency, low variance - the proportion of off-targets that have high efficiency efficiency but low variance;
7. low efficiency, high variance - the proportion of off-targets that have low efficiency but high variance;

Supplementary Table 7: Performance of the models on cross-validation

This supplementary table contains information about predictive performance of the trained models on 10-fold cross-validation. The columns are as follows:

1. Index column - name of the model which consists of dataset name, used architecture (C for CNN, R for RNN) and loss function (E for ELBO, E+M for ELBO+MSE);
2. 0.68, 0.95, 0.997 - values of p_{68}, p_{95} and $p_{99.7}$;
3. PCC and PCC-pval - Pearson Correlation Coefficient with respective p-value;
4. SCC and SCC-pval - Spearman Correlation Coefficient with respective p-value;
5. rsquared - values of r^2.
